# Blocking the FKBP12 induced dendrimeric burst in aberrant aggregation of α-synuclein by using the ElteN378 synthetic inhibitor

**DOI:** 10.1080/14756366.2019.1667342

**Published:** 2019-09-24

**Authors:** Gabriella Caminati, Maria Raffaella Martina, Stefano Menichetti, Piero Procacci

**Affiliations:** a Department of Chemistry “Ugo Schiff”, University of Florence, Sesto Fiorentino, Italy;; b Center for Colloid and Surface Science (CSGI), University of Florence, Sesto Fiorentino, Italy

**Keywords:** FKBP12 inhibitor, α-synuclein, Parkinson’s disease, amyloid aggregation, PD drug

## Abstract

α-Synuclein (α-syn), a disordered cytoplasmatic protein, plays a fundamental role in the pathogenesis of Parkinson’s disease (PD). Here, we have shown, using photophysical measurements, that addition of FKBP12 to α-syn solutions, dramatically accelerates protein aggregation, leading to an explosion of dendritic structures revealed by fluorescence and phase-contrast microscopy. We have further demonstrated that this aberrant α-syn aggregation can be blocked using a recently discovered non-immunosuppressive synthetic inhibitor of FKBP12, ElteN378. The role of FKBP12 and of ElteN378 in the α-syn aggregation mechanism has been elucidated using molecular dynamics simulations based on an effective coarse-grained model. The reported data not only reveal a new potent synthetic drug as a candidate for early stage treatment of α-syn dependent neurodegenerations but also pave the way to a deeper understanding of the mechanism of action of FKBP12 on α-syn oligomeric aggregation, a topic which is still controversial.

## Introduction

The plastic α-syn protein is a key player in the pathogenesis of Parkinson’s disease (PD)^1–3^. In pathological conditions, this protein is present in the brain cells in fibrillar, aggregated forms called Lewy bodies. Members of the FK506-binding protein (FKBP) family were recently shown to be implicated in neurological disorders and structure formation^1^^,^^3^^,^^4^. The involvement of FKBP12 in the pathogenesis of α-syn-dependent neurodegeneration has been repeatedly shown in post-mortem studies on the brain of patients with neurodegenerative diseases^1^^,^^5^^,^^6^. Recent investigations revealed an unbalance of the endogenous FKBP12 concentration in the very early stages of PD^4^, as well as the presence of FKBP12 in Lewy bodies^6^. Beeklandt and coworkers^7^ proposed that proteins of the FKBP family, and notably FKBP12, induce a faster aggregation of amyloid α-syn, by binding α-syn via the prolyl peptidyl isomerase domain^8^ to one of the proline in the hydrophilic C-terminus of the monomer^9^. Despite the cited mounting evidence about a possible role for FKBP12 in synucleopathies and neurological disorders through the enhancement of α-syn aggregation, the mechanism of such enhancement has not been unraveled yet.

**Figure 1.**
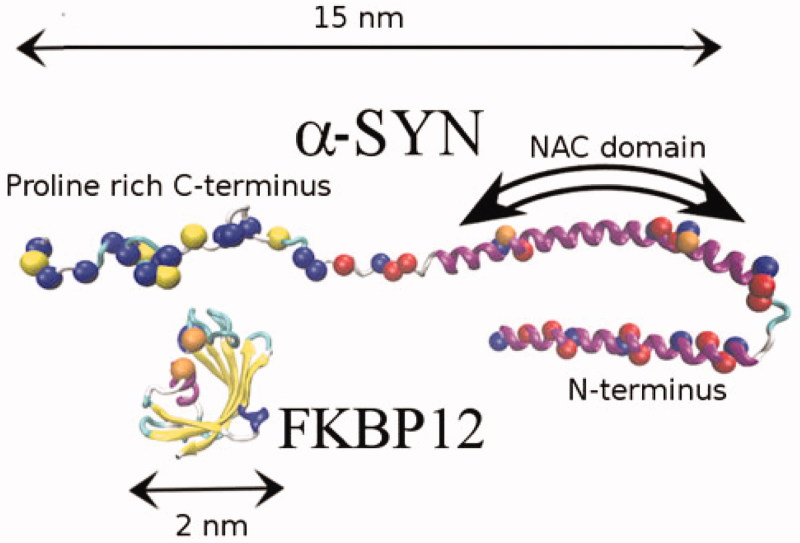
α-syn and FKBP12 structures. Red and blue balls on α-SYN indicate negatively and positively charged residues, respectively. Proline is indicated in yellow colour.

In Figure 1, we show a ribbon representation of α-syn monomer along with the FKBP12 protein. The α-syn monomer primary structure comprises three distinct segments. The C terminus (92–140) is a polar coil region bearing a strong negative charge (−15**e**) and hosting all five prolines in α-syn. The equally polar 1–40 N α-helical^10^ terminus has a mixture of negatively and positively charged residues with a slight prevalence of the latter (+3**e**). The central NAC domain, including amino-acids 40 to 92, is characterised by a sequence of mostly hydrophobic residues and is involved in β-sheet formation^11^. Several studies have shown that α-syn fibrils are characterised by a β-rich hydrophobic solvent protected core^12–15^ with external hydrophilic filaments due to the C and N termini.

Using a combined approach of experimental measurements and molecular dynamics simulations, in this contribution we aim at elucidating the role of FKBP12 in α-syn aggregation kinetics and aggregate morphology suggesting at the same time a possible therapeutic agent, the synthetic ElteN378 FKBP12 inhibitor^16^, in PD neuropathies. ElteN378 is a low atomic weight FKBP12 ligand with affinity comparable to that of the natural binder FK506. This ligand is affordable from commercially available precursors and, as shown in Supplementary Figure S1 in Supporting Information, has been designed to optimally expose the two contiguous carbonyl oxygen atoms in the proline-mimetic chain for H-bond-driven FKBP12 docking^16^.

Aggregation of α-syn was studied *in vitro* following the change in the photophysical behaviour of the fluorescent probe ThT in three different systems: α-syn, α-syn/FKBP12 1:1 and α-syn/FKBP12 1:1 inhibited by ElteN378. Imaging of mature aggregates of the previous systems was obtained with fluorescence and phase-contrast microscopy. The collected experimental evidence on aggregation kinetics and morphology was interpreted using extensive MD simulations based on a coarse-grained model (CG) for α-syn and α-syn-bound FKBP12 monomers with inter- and intra-molecular interactions driven by a potential mean force effectively accounting for the amphiphilic nature of the α-syn monomer. The results showed striking similarities between the structures obtained from the CG simulations and the experimentally observed morphology of the aggregates suggesting that the mechanism and fate of the aggregation process can be dramatically modified by altering the balance between hydrophilic and hydrophobic forces.

## Aggregation kinetics

Unseeded samples were incubated at 37 °C in glass vials under gentle constant stirring^17^. We used 1 µM concentration for both α-syn and FKBP12 in Tris buffer. The physiological cytosolic concentration of α-syn is brain cells was reported to be^18^ in the low micromolar range and is probably even below this level^19^. On the other hand, FKBP12 is especially abundant in brain^20^ with physiological concentrations reaching 3 µM, according to recent studies^21^.

Aliquots of the incubated solutions were withdrawn and characterised at different time intervals for 20 weeks. ThT was added only before each measurement to avoid probe-induced artifacts on aggregation. At the working ThT concentration used in this work (3 µM), the probe is present in solution exclusively as monomer and the corresponding emission spectrum shows a single weak emission band centred at 445 nm (Supplementary Figure S4a) upon excitation at 350 nm, as commonly found^22^^,^^23^ for ThT monomer in protic solvents. Control experiments exclude ThT interaction with native α-syn or with the globular FKBP12, in agreement with literature reports^24^. Further details on the experimental procedures are reported in Supporting Information.

**Figure 2.**
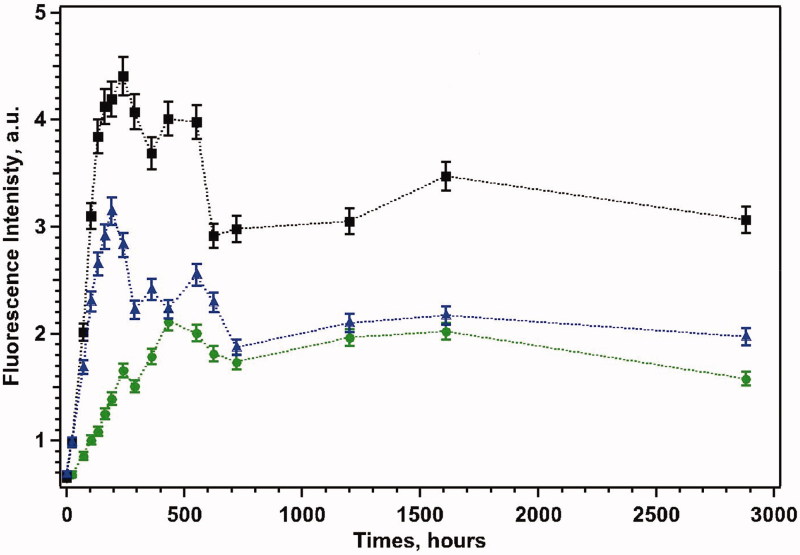
Fluorescence emission intensity at λ = 480 nm for α-syn (green circles), α-syn/FKBP12 (black squares) and α-syn/FKBP12/ElteN378 (blue triangles) systems. For each system, the reported error bars have been computed by analysing the ThT fluorescence intensity measured in three independent samples (see Supporting Information for further details).

When ThT was added to α-syn solutions we observed a significant change in the position and the intensity of the excitation and emission bands: in Figure 2, we report the ThT fluorescence intensity at λ_em _=480 nm with λ_exc _=410 nm as a function of time for all examined systems. For free α-syn, in the absence of any added seed, ThT emission intensity changes with time following the well-known sigmoidal curve^23^^,^^25^ although the lag phase is contracted in this time scale. The curve is consistent with a nucleation-dependent elongation model of fibril formation^26^^,^^27^. After 18 days, aggregation proceeds through elongation of the protofibrils without further change in ThT emission. In the equimolar α-syn-FKBP12 system, fluorescence emission increases at a faster rate reaching larger intensity values at a much earlier stage (10 days), before decreasing to a plateau value.

Addition of 200 nM ElteN378 inhibitor in the FKBP12-α-syn system results in a similar kinetic profile but with a reduction of the aggregated phase, followed, once more, by a decrease in ThT signal. While rarely reported in conventional aggregation studies^28^, the decrease in ThT fluorescence in aged α-syn containing solutions, consistently detected in all analysed independent samples can be explained by a combination of chemical-physical effects that include (i) redistribution of the ThT probe in other sites of the growing oligomers characterised by a shift of the maximum of ThT emission band, (ii) formation of mature aggregates with different morphology and exposed β-sheet content (interconverting structures), and (iii) partial loss of emission intensity due to deposition of fibrillar tangles. The stabilisation of the ThT fluorescence signal in long term measurements allows for the exclusion of progressive sedimentation of large aggregates.

In order to elucidate the observed aggregation kinetics, we have done extensive MD simulations based on an elementary CG model for α-syn and α-syn-bound FKBP12 using the program ORAC^29^^,^^30^. Computational details on the CG approach are provided in Supporting Information. Briefly, we adopt a 15 beads CG representation of the monomeric α-syn chain, made up of three distinct parts of five beads length each: a central hydrophobic part representing the NAC domain and two terminal hydrophilic parts. The five beads approach is based on a recent model for the α-syn mature fibrils based on a five strands monomer^11^^,^^31^^,^^32^. We use a solvent-free model with renormalised bead-bead non-bonded interactions so as to mimic, in a water environment, the aggregation of hydrophobic moieties and solubilisation of the hydrophilic groups. We further assume that, when proline-bound to the C terminus of α-syn, the globular protein FKBP12 exposes an unsaturated β-strand acting as a seed for the binding of the β-sheet forming NAC domain, as illustrated in Figure 1.

**Figure 3.**
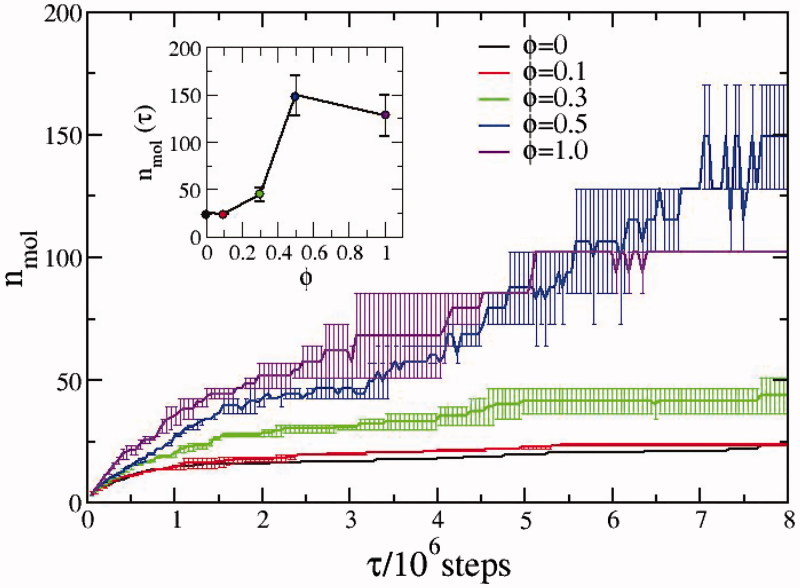
Aggregation kinetics measured by the average number of monomers composing the aggregates for various fractions ϕ (see text) of FKBP12-bound α-syn. The total number of the 15-beads α-syn monomers is in all case equal to 512. For each fraction, the errors have been evaluated by averaging the data over three independent simulations. In the inset, we report the average number of monomers composing the aggregates in the final configuration.

The FKBP12 addition at the onset of α-syn aggregation is hence modelled by simply replacing one of the terminal hydrophilic beads with a hydrophobic one in a fraction, say ϕ, of α-syn monomers computed according to the nominal concentration of the species and to the equilibrium dissociation constant for the FKBP12-α-syn complex estimated to be in the micromolar range (see Supporting Information). The effect of the tight-binding ElteN378 inhibitor in the α-syn-FKBP12 solution is simply that of reducing the FKBP12-effective concentration in a strictly dose-dependent manner. In Figure 3, we show the calculated CG kinetics for the pure α-syn and for samples with different fractions ϕ of FKBP12-modified α-syn monomers. Inspection of Figure 3 shows that the kinetics is accelerated with increasing fraction of FKBP12-modified α-syn molecules. Correspondingly, the final stationary of value of the average number *n*_mol_ grows with the fraction of FKBP12-bound α-syn monomers, ending up to only few supramolecular macro-aggregates for ϕ>0.5. In the inset, we report the final value of *n*_mol_ as a function of ϕ evidencing the non-linear effect of FKBP12 addition. Up to ϕ = 0:3, the aggregation kinetics resembles that observed in the sample of pure α-syn. Above the threshold fraction of ϕ > 0.3, the aggregation kinetics undergoes an abrupt regime cross-over, exhibiting a much faster growth

## Aggregation morphology

**Figure 4.**
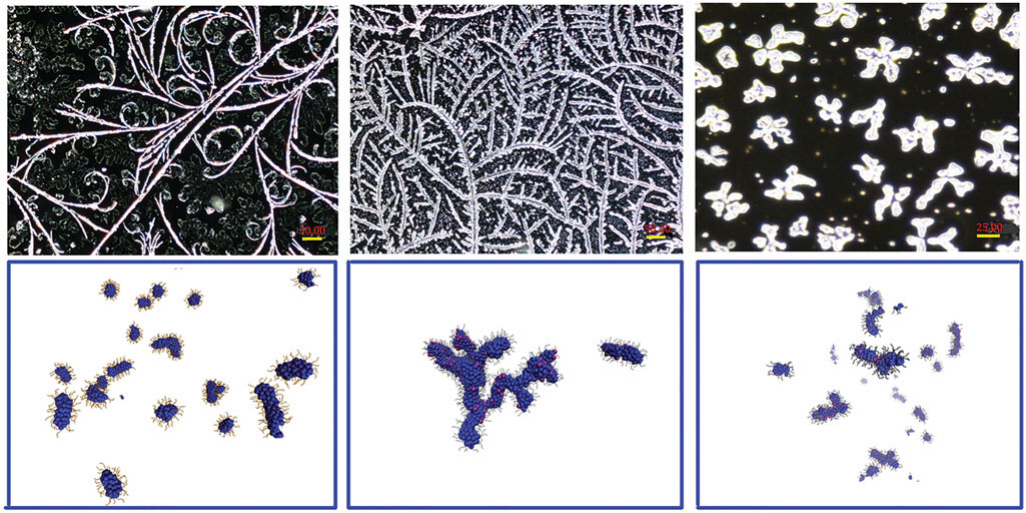
Upper panel: fluorescence images of mature fibrillar aggregates. Left: aggregated pure α-syn. Central: dendritic aggregated structures for equimolar mixtures of FKBP12/α-syn. Right: small cross-like or star-like amorphous structures of the three components system: ElteN378/FKBP12/α-syn. Lower panel snapshots of three MD simulations based on a CG model: α-syn alone (left), α-syn/FKBP12 mixture (central) and ElteN378/FKBP12/α-syn mixture (right). Hydrophobic beads are shown as blue spheres. FKBP12-bound beads are shown as red spheres. Hydrophilic N- and C-terminus beads are shown in orange bond representation.

Aliquots of the mature incubated solutions were imaged by means of fluorescence and phase-contrast microscopy. Typical fluorescence images, consistently observed in all independent samples, are reported in Figure 4 while typical phase contrast results are shown in Supplementary Figure S2. As shown in the upper panel left image of Figure 4, images of α-syn at very long aggregation times show the presence of a high concentration of linear structures as found also by other authors^14^.

The linear morphology of the fibril in α-syn aggregation has been rationalised^32^^,^^33^ in terms of an in-register parallel β-sheet structure with systematic stacking of the hydrophobic NAC domains of the monomers along the fibril axis. The central upper panel of Figure 4 shows images of samples taken from an equimolar solution of FKBP12-α-syn. The observed aggregation pattern is dramatically different, exhibiting an impressive dendritic explosion of highly branched structures. Remarkably, such quasi-fractal growth is strongly reduced when ElteN378 is present in the α-syn/FKBP12 system: the number of branching nodes is drastically scaled down resulting in much smaller and less abundant aggregates with a cross-like morphology. The rational for the observed behaviour lies in the strong binding affinity of ElteN378 for FKBP12, previously reported by us^16^. The presence of the tightly bound ElteN378 in the FKBP12 substrate pocket prevents the possibility of FKBP12 to interact with the proline-rich C terminus of α-syn. This translates in a reduction of the concentration of “active” FKBP12 seeds for explosive branching of α-syn aggregates.

The lower panel of Figure 4 refers to the end states of the CG simulations for different fraction of FKBP12-doped α-syn. In striking correspondence with the experimental observations, the sample containing only α-syn (ϕ=0) spontaneously aggregates in linear fibrils exposing the hydrophilic filaments towards the solvent, roughly perpendicularly to the fibril axis. When one-third of the α-syn monomers are FKBP12-doped (ϕ**=**0.3), the final supramolecular aggregates are poorly branched (right lower panel). The same morphology is observed experimentally in the ElteN378-FKBP12 α-syn mixture. As above outlined, when ϕ**>**0.3, the kinetics undergoes a drastic change, with the sample ending up in forming few highly branched macro-aggregates (central lower panel). In Supporting Information, we estimate that in the equimolar FKBP12-α-syn solution the fraction α-syn monomers bearing a bound FKBP12 is in the range ϕ**=**0.3:0.4. The estimate is based on the measure of the fluorescence quenching of the strong tryptophan emission band at 300–350 nm occurring in FKBP12 solutions upon ligand addition. The presence of the tight-binding inhibitor ElteN378 in FKBP12-α-syn mixture lowers this fraction below 0.3, corresponding to the value for the cross-over regime reported in the inset of Figure 3).

## Conclusions

We have shown using ThT fluorescence and microscopy, that FKBP12 and α-syn in a 1:1 molar ratio spontaneously aggregate forming a dendritic explosion of aggregated structures. This process can be tamed, in a dose-dependent manner, using the non-immunosuppressive ElteN378 synthetic inhibitor. CG simulations showed that the aggregation of α-syn in linear fibrils as well as in complex branched supramolecular aggregates is elicited by the hydrophobic aggregation of the central non-polar NAC residues of the monomeric unit. Remarkably, our rudimental CG model for the α-syn monomer was able to reproduce the essential morphological features of the supramolecular aggregates in the final stages with no necessity of introducing any detail on the secondary structure, therefore hinting that a continuous and variegate β-reorganisation process follows the primary event of hydrophobic-driven association of the NAC domains in oligomers.

## Supplementary Material

Supplemental Material

Supplemental Material

Supplemental Material

Supplemental Material
