# Supplementary material for: Blocking the FKBP12 induced dendrimeric burst in aberrant aggregation of α-synuclein by using the ElteN378 synthetic inhibitor
Source: J Enzyme Inhib Med Chem. 2019 Sep 24;34(1):1711–5. doi: 10.1080/14756366.2019.1667342 (PMC6764402; doi:10.1080/14756366.2019.1667342)
Supplement: Supplemental Material [file IENZ_A_1667342_SM9945.pdf]

Supporting Information fo "Blocking the FKBP12 induced dendrimeric burst in aberrant aggregation of α-synuclein by using the synthetic ElteN378 inhibitor"

Gabriella Caminati,[*,†,‡] Maria Raffaella Martina ,[†,‡] Stefano Menichetti,[†] and Piero Procacci[*,†]

†*Department of Chemistry "Ugo Schiff" , University of Florence, Via della Lastruccia 3-13, Sesto Fiorentino, I-50019 Italy*

‡*Center for Colloid and Surface Science (CSGI), University of Florence, della della Lastruccia 3-13, Sesto Fiorentino, I-50019 Italy*

E-mail: gabriella.caminati@unifi.it; procacci@unifi.it

Materials and Methods

Materials

α-synuclein human recombinant (α-syn) expressed in E. coli (MW 14460) was obtained from rPeptide. Stock solution was prepared in Tris 10 mM/HCl buffer (pH 7.4, 0.15 M NaCl) at concentration of 1 mg/ml. Protein concentration was determined measuring the absorption spectra with a Lambda900 spectrophotometer (Perkin Elmer) using $\epsilon_{275} = 5600$ M^{-1} cm^{-1}. FK506 Binding Protein (FKBP12) expressed in E. coli (MW 11900) was supplied by CIRMMP (Italy, Florence). The purity of FKBP12 was greater than 95% determined by

SDS electrophoresis. The concentration of FKBP12 in Tris buffer was determined by UV absorbance at 280 nm using $\epsilon_{280} = 9970$ M^{-1}. ElteN378 was synthesized as reported in Ref.[1] In Figure S1, we show the chemical structure of the chiral ElteN378 (on the right) along with the structure of the ElteN378 bound to a FKBP12 protein (on the left).[1] The design of ElteN378 is such that the two carbonyl oxygen atoms (highlighted in the chemical structure of Figure S1) can make two H-bonds with the hydrogen atom of the hydroxy group in Tyr82 (PDB atom label HH) and the amide hydrogen atom NH of Ile56 of the FKBP12 protein, while the two non polar phenyl moiety interact via $\pi - \pi$ stacking.

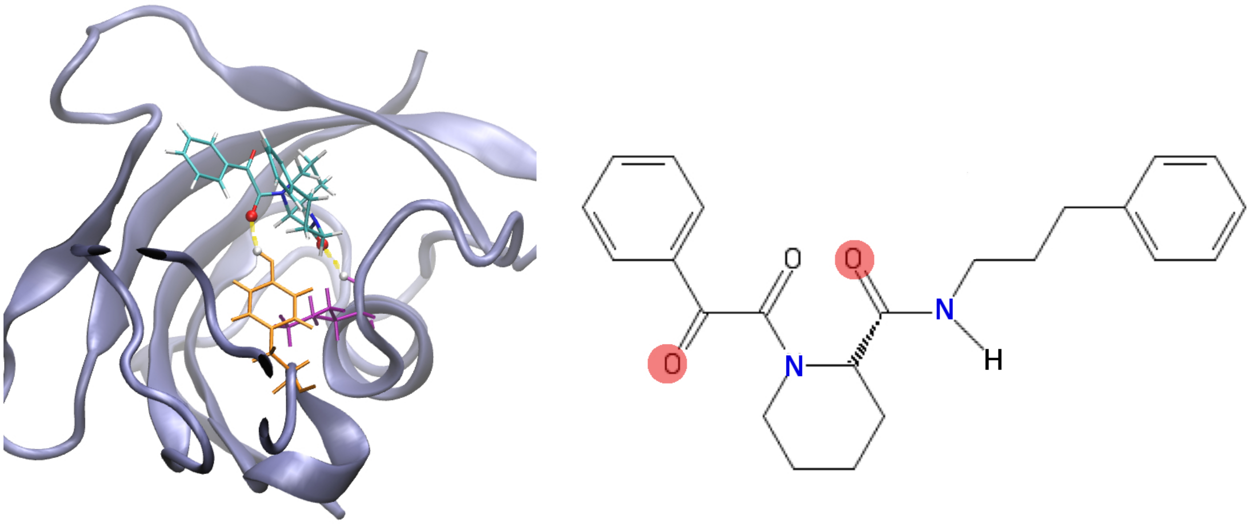

Figure S1: Right: chemical structure of ElteN378. Left: structure of the complex ElteN378 and FKBP12 taken from Ref.[1] Ile56 and Tyr82 are shown in orange and magenta colors, respectively. H-bond between HH(Tyr82)-O2(ElteN378) and HN(Ile56)-O3(ElteN378) are highlighted in yellow.

Thioflavine T (ThT) was purchased from Sigma-Aldrich Chemie GmbH (Schnelldorf, Germany). Tris 10 mM/HCl buffer was prepared with Trizma®base, Trizma®hydrochloride and NaCl purchased from Aldrich and used as received. All solutions were prepared with

deionized water (resistivity =18 MΩ cm, pH = 5.6 at 20 °C) obtained from a Milli-RO coupled with a Milli-Q set up (Millipore, Italy).

Photo-physical investigation of aggregation kinetics

Fibrillation was achieved by incubating 1 μM monomeric α-syn stock solutions in glass vials at 37° under constant stirring. Three sets of independent samples were prepared with the same concentration of α-syn. Appropriate amount of FKBP12 was added to two series of samples to reach a final concentration of 1 μM whereas the FKBP12 inhibitor, ElteN378, was added only to one of the three sample lines to reach a final concentration of 200 nM.[1] Aliquots of the incubated solutions were withdrawn and characterized at different time intervals. The fluorescent probes were added only 30 min before each measurements. This time interval was found to ensure equilibrium distribution of the probes in separate experiments as a function of probe incubation time. Final concentration of ThT in all samples was 3×10^{-6} M.

Except for the moderate agitation, which is known to greatly accelerate the diffusion-limited aggregration process, these experimental conditions were chosen in order to observe aggregation phenomena using physiological protein concentration[2–4] and pH levels. In this regard, it should be mentioned that in many biochemical *in vitro* studies of α-syn aggregation, high levels of α-syn monomer (40-200 μM) are commonly used and the process is often seeded with polyamines or fibril fragments. With such experimental setup, aggregation occurs very quickly, producing mature fibrils in just 20-40 h. The significance of these kind of studies for the aggregation kinetics of α-syn in physiological conditions (and in PD etiology in general) is unclear.

Fluorescence spectra were recorded on a LS50B spectrofluorimeter (Perkin-Elmer). Excitation and emission slits were set to 10 nm. QS cell with 0.3 cm optical path length from Hellma (Hellma GmbH & Co. KG, Muellheim, Germany) were used for fluorescence experiments, whereas 1 cm OP cuvettes were used for absorption measurements. Cuvettes were cleaned with piranha solution and carefully rinsed with water and ethanol. The cuvettes

were dried by nitrogen flushing prior to each measurement. All fluorescence measurements were run at 20 °C unless otherwise stated. The emission spectra of the corresponding blank solutions were always recorded separately and subtracted. Emission spectra of the samples containing the ThT probe were obtained exciting in the 300-450 nm range. Emission-dependent excitation spectra were acquired for emission wavelength in the range 430-500 nm. Reported data represent the average of at least three independent experiments.

Imaging of mature fibrillar aggregates

Images of 90-days old samples were taken with a Diaphot 300 Inverted microscope (Nikon) equipped with a LWD condenser capable of phase contrast, differential interference and bright field microscopy. The instrument was provided also with an epi-fluorescence attachment module (TMD-EF, Nikon) with a 100W Epi Fluorescence HBO mercury arc lamp house and power supply, an Epi-Fluorescence beam delivery system with selectable filters cassettes installed. Epi-fluorescence images were taken with different filters combination to selectively isolate the 450 nm emitting species and the 480 n emitting ThT species. In the images reported in the paper we used the BV combination (IF 400-450 excitation filter, with main excitation wavelength 436 nm, plus a DM455 dichroic mirror). Both 20x and 40x objectives were used to explore the homogeneity of the samples, scale bars are reported in the images accordingly. Aliquots withdrawn from the samples after 90 days of incubation where imaged immediately after withdrawal with and without addition of ThT to the samples. We applied 5 to 10 μl of this sample onto a microscope glass slide covered with a cover-slip at a distance of ca. 15 μm (as measured by z-scanning the samples with a Leica Confocal Laser Scanning Microscope). Samples were prepared following procedures already described[5] that includes proper sealing to avoid solvent evaporation either with enamel or wax (recipe from Nikon Imaging Center) to prevent evaporation. Similar results were also obtained using closed chambers (Nunc™Lab-Tek™II Chambered Coverglass with borosilicate glass bottom, 8 well chambers). Epi-fluorescence images are shown in the main text (Figure 4) whereas

phase contrast microscopy results are reported in Figure S2.

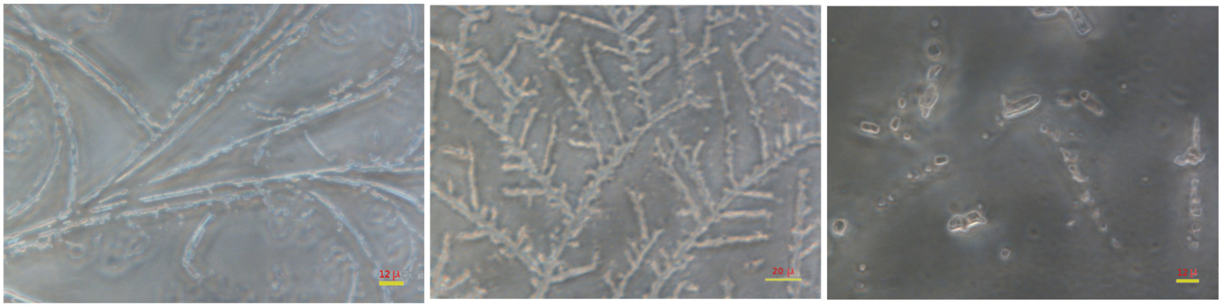

Figure S2: Phase contrast images of aged samples of 1 μM of α-syn (left), 1 μM of α-syn:FKBP12 (center) and 1 μM of α-syn:FKBP12 and 0.2 μM of ElteN378 (right).

Simulation procedures

The α-syn monomer chain is represented in our coarse-grained (CG) approach by 15 beads, made up of three distinct part of five beads length each, i.e. a central hydrophobic part (corresponding to the NAC domain) and two terminal hydrophilic parts. As shown in Figure S3, in the monomer, the CG beads have hence only two possible colors, namely hydrophilic (terminal segments 1-5 and 11-15) and hydrophobic (central segment 6-10). Consecutive beads are bound via a stretching harmonic potential with $r_0 = 1.5$ nm equilibrium distance and with a stiff force constant of 230 in units of $RT \times \mathrm{nm}^{-2}$. A harmonic bending potential with equilibrium angle α =120° and force constant $k = 230$ in units of $RT \times \mathrm{rad}^{-2}$ is enforced between three consecutive beads, irrespective of their color. The equilibrium bead-bead distance and bending angle are chosen so that the 15 beads CG α-syn monomer, when in the fully extended state, has a length of the order of 20 nm. We use a solvent free model with re-normalized bead-bead non-bonded interactions so as to mimic, in a water environment, the aggregation of hydrophobic moieties and solubilization of the hydrophilic groups. To this end, a strongly repulsive atom-atom potential is assigned to the hydrophilic beads (1-5 and 11-15) while an attractive atom-atom potential is used to model the interactions of the hydrophobic NAC domain central beads. The potential functional form for the bead-

bead non bonded interaction is of the Lennard-Jones type and standard Lorentz-Berthelot mixing rules applies for the hydrophilic-hydrophobic interactions. The Lennard-Jones ϵ well depth and σ parameters for hydrophobic-hydrophobic interactions are tuned so as to obtain, at a distance of $\simeq$ 1.5 nm, approximately a gain of 10 RT units per bead (representing approximately 10 residues), corresponding to a reasonable mean value of 1 RT units gain for the aggregation of two hydrophobic residues in water.[6] Potential parameters are reported in the Figure S3 that exemplifies the CG model used for α-syn and α-syn/FKBP12 mixtures.

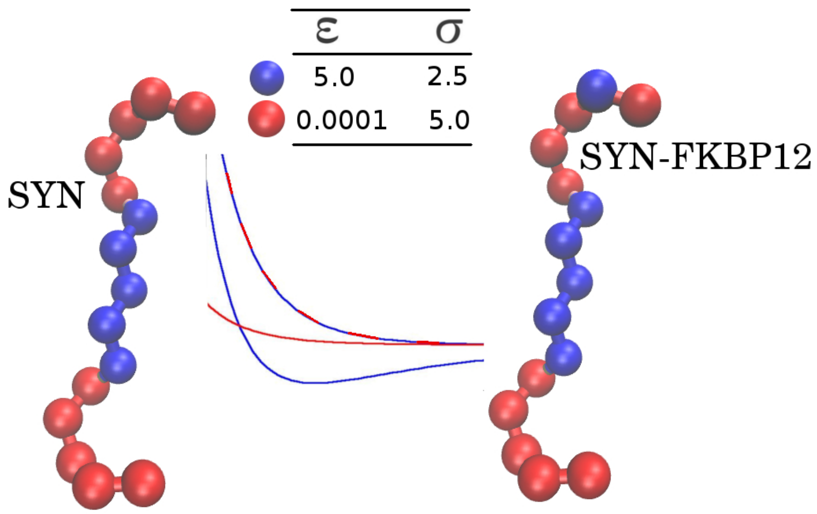

Figure S3: Coarse grained model for α-syn and α-syn-FKBP12 complex. Hydrophobic and hydrophilic beads are in blue and red, respectively. The ϵ and σ Lennard-Jones parameter are in RT units and nm respectively.

At the start of the simulation, 512 α-syn monomeric chains are inserted randomly in a cubic box of 300 nm side length (200 bead units) corresponding to an initial volume of 2.7×10^7 nm^3, yielding a volume per molecule 5×10^4 nm^3 corresponding to an initial concentration of $\simeq 30\mu$M. Periodic boundary conditions (PBC) are used. At ordinary temperature, the *in vacuo* diffusivity of a molecule is about four to five order of magnitude faster with respect to that typical of the liquid water solution. The aggregation rate of the α-syn monomers

depends on the diffusivity and on the α-syn initial concentration.

Given the initial concentration of monomers of $\simeq 30\mu$M, setting T=300 K and re-scaling the mass of the bead to 4 Da (yielding a monomer mass of 60 Da instead of the actual 14.46 kDA), we expect, in our CG model, aggregation phenomena to occur in few millions of time steps. α-syn-FKBP12 mixtures are obtained by replacing one of the terminal hydrophilic beads with an hydrophobic one in an appropriate fraction of α-syn molecules. The effect of the tight-binding ElteN378 inhibitor in the α-syn-FKBP12 mixture is simply that of reducing the FKBP12 effective concentration in a strictly dose dependent manner.

Starting from the 512 CG system with 15 beads per monomer (7680 “atoms” system), we therefore performed 8 millions of time steps long molecular dynamics simulation for α-syn alone (Figure S3 left) and four equivalent runs where a fraction of 0.10, 0.30, 0.5 and 1.0 of the 512 monomers were modified by replacement of a hydrophilic bead with a hydrophobic one the termini (Figure S3 right). The starting configurations referring to the fraction α-syn-FKBP12(0.1,0.3,0.5,1.0) were obtained by modifying, according to the scheme of Figure S3, 56, 152, 256 and 512 random molecules of α-syn. Each of these 5 series of simulations was performed in triplicates for error assessment. All simulations were carried on at constant volume and constant temperature. Temperature was held constant to 300 K using a Nosé-Hoover thermostat. The equations of motion were integrated using a multiple time step algorithm with the longest step corresponding to about $\tau = 1 \times 10^{-4} t_c$ where $t_c = V/(\pi\sigma^2 n_b)(m_b/k_B T)^{1/2}$ is defined as the hypothetical mean collision time between free hydrophobic beads with V, σ, m_b and n_b being the volume of the MD box, the van der Waals radius of the hydrophobic bead, the mass of the bead the total number of beads, respectively. The cutoff for the non bonded interactions was set to 50 nm. All simulations were done using the program ORAC.[7,8]

We stress here that, since the CG driven aggregation process is diffusion limited, the final aggregation pattern for the various fractions of FKBP12-doped α-syn does not change if one choose an initial concentration of 1 μM (data not shown) as in the experimental setup.

However, as t_c depends linearly on the volume, the length of the simulation to observe the final aggregation state should be extended up to at least 200 millions of time steps. The supporting material includes the CG topological and parameter file and ORAC[9] input examples for free synuclein and for FKBP12-doped synuclein, as well as instructions for reproducing the CG simulations at C=30 μM or at C=1 μM of α-syn.

Fluorescence spectra of ThT 3 μM in Tris Buffer

ThT emission and excitation spectra in buffer solution (shown in the Figure S4) were recorded both at the working concentration (3 μM) and at concentrations higher than the reported c.m.c (50 μM). When ThT concentration is increased beyond the reported c.m.c,[10] a new broad and unstructured band at 480 nm is clearly evident.

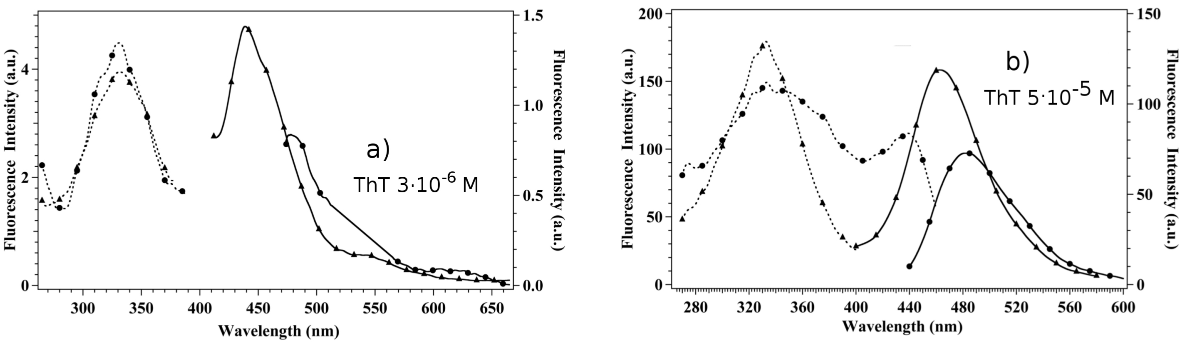

Figure S4: Excitation (left) and emission (right) spectra of ThT 3 μM (a) and 50 μM (b) in Tris Buffer Solution. Solid lines are the emission spectra recorded upon excitation at 350 nm (triangles) and 440 nm (circles). The dashed curves are the excitation spectra recorded at 450 nm (Triangles) and 482 nm (circles).

Fluorescence spectra of ThT 3 μM in α-syn containing systems

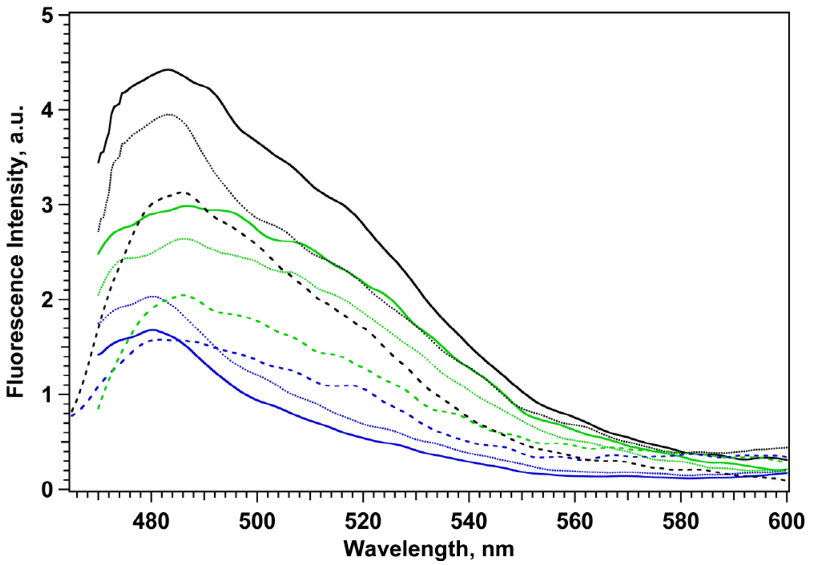

Figure S5: Representative fluorescence emission spectra of ThT after 10 (solid line), 26 (dashed line) and 120 (dotted line) days of incubation in α-syn (blue curves), α-syn/FKBP12 (black curves) and α-syn/FKBP12/ElteN378 (green curves) systems, $\lambda_{exc} = 410$ nm.)

Aggregation process from CG simulations

In the MPEG files provided in the Supporting information, the evolution the systems, for increasing fraction of FKBP12-doped α syn monomers, can be followed during the course of the CG simulations. The beads on the terminal hydrophilic segments of the monomers are in bond representations, while the central hydrophobic beads are the blue spheres. An FKBP12 unit replacing one bead on the hydrophilic segments is represented as a red sphere. In striking correspondence with the experimental observations (see main text), the sample containing only α-syn ($\phi = 0.0$, file `NVT0.0.mpg`) spontaneously aggregates in linear fibrils exposing the hydrophilic filaments pointing towards the solvent roughly perpendicularly to the fibril axis. In the sample containing a fraction $\phi = 0.3$ of FKBP12-doped monomers (file `NVT0.3.mpg`), the kinetics undergoes only a mild acceleration. In these conditions, the

final supramolecular aggregates are mostly linear or poorly branched, a situation resembling that observed experimentally upon addition of only 200 nM of ElteN378 to a 1μM solution of the equimolar FKBP12 α-syn mixture. As above outlined, when $\phi > 0.3$, the kinetics undergoes a drastic change, with the sample ending up in forming few highly branched macro-aggregates file `NVT0.5.mpg`. The formation of highly branched dendrimeric structures is observed experimentally in the 1:1 α-syn FKBP12 1 μM solution, i.e. when no fraction of the available FKBP12 is made inert by ElteN378. On the overall, the reported simulation data appears to confirm that the aggregation in linear fibrils of α-syn is elicited by the hydrophobic aggregation of the central non polar residues of the monomeric unit. Remarkably, our simple CG model for the α-syn monomer appears to capture the essential morphological features of the supramolecular aggregates in the final stages with no necessity of introducing any detail on the secondary structure. The C and N terminus, as observed in Ref.,[11] remain disordered and solvent exposed during the aggregation process. According to our simulation, FKBP12, by binding proline on these solvent exposed terminal filaments, provides a new seed for branching on the main growing fibril axis.

Binding affinity of α-syn for FKBP12 and cross-over regime in aged samples

Besides rationalizing the aggregation of α-syn in terms of elementary thermodynamic forces, the simulations data with the reduced CG model provide a valuable corresponding state approach to explain the experimental observations in the FKBP12-α-syn mixture along with the effect of the ElteN378 tight binding ligand. We recall that in the experimental condition we have a 1:1 FKBP12-α-syn 1μM solution. The dissociation constant for the FKBP12-α-syn constant is not known. As stated in the manuscript, binding of α-syn monomer to FKBP12 is likely to involve the proline rich C terminus region. Proline mimetic FKBP12

inhibitors with binding pyrrolidine moieties are known to be much less potent than pipecolic compounds like sb3[12] or Elte,[1,13] exhibiting dissociation constant typically in the *micromolar* range.[14] An estimate of the α-syn(monomer) binding affinity for FKBP12 has been obtained by measuring the tryptophan fluorescence quenching of the strong emission band at 300-350 nm, occurring in FKBP12 solutions upon ligand addition. In Figure S6 we show the fluorescence in the 300-350 nm region for FKBP12 0.74 μM in Tris buffer and for FKBP12 mixtures (Tris buffer) containing i) ElteN378 0.20 μM, ii) α-syn 1.1 μM, and iii) α-syn 1.1 μM and ElteN378 0.20 μM. α-syn-FKBP12 and α-syn-FKBP12-ElteN378 using freshly prepared stock solutions of α-syn and by recording the spectra. The emission spectra of the

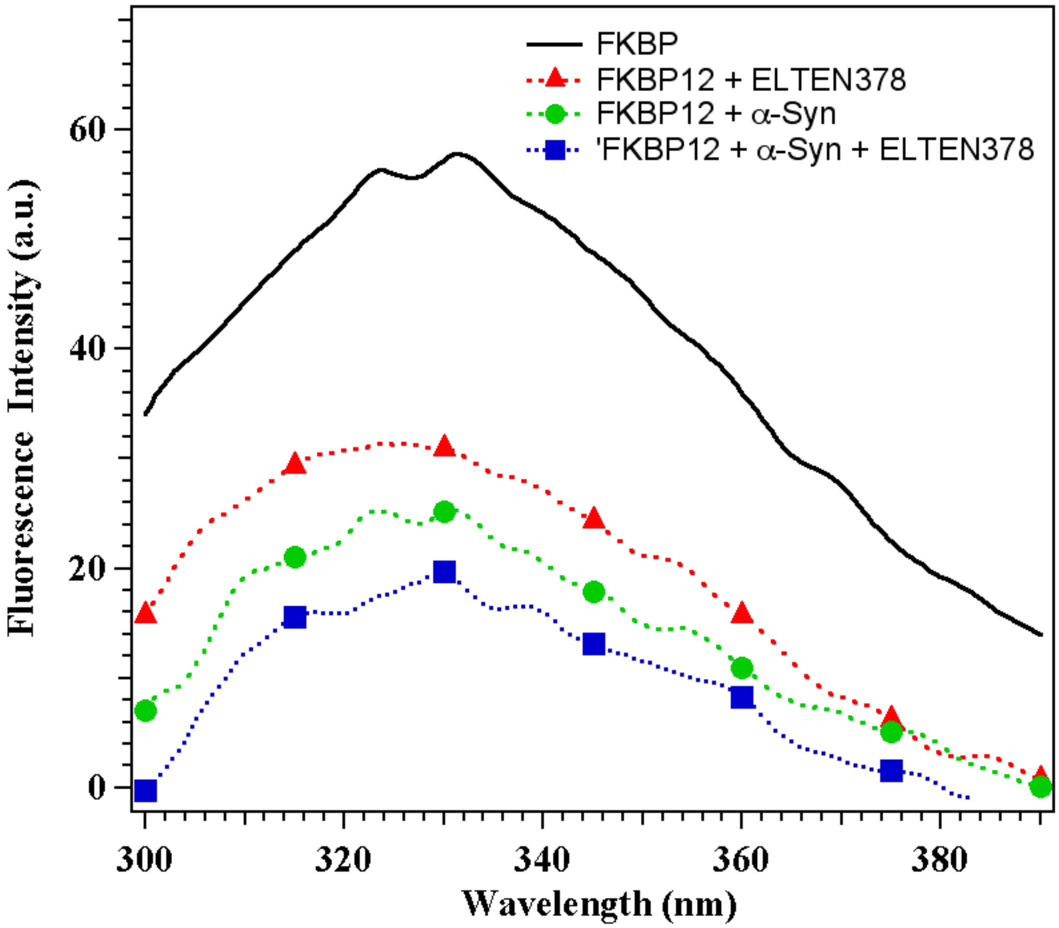

Figure S6: Fluorescence intensity at λ_{em} = 330 nm (λ_{exc} = 280 nm) of FKBP12 (solid line, C=0.74 μM) in presence of 0.20 μM ElteN378 (green dotted line), of 1.1 μM α-syn (black dotted line) and in presence of 1.1 μM α-syn and 0.2 μM ElteN378 (blue dotted line).

corresponding blank solutions (ElteN378, α-syn and α-syn+ElteN379) were always recorded and subtracted. From the knowledge of the dissociation constant of ElteN378 (K_d = 3 nm),[1] the observed emission intensity ratios at $\lambda_{\rm rmexc} = 330$ nm, which are proportional to the fraction of bound FKBP12, allows to estimate the dissociation for complex FKBP12-α-syn as $K_d = 1.0 : 1.5$ μM.

The dissociation constant for the FKBP12-α-syn complex in presence of the tight binding inhibitor ElteN378 is given by

$$\frac{K_d}{C} = \frac{\frac{(C - C_{\rm E} - [{\rm FS}])}{C}\frac{(C - [{\rm FS}])}{C}}{\frac{[{\rm FS}]}{C}} \tag{1}$$

where C is the concentration of the equimolar FKBP12 α-syn mixture, $C_{\rm E}$ is the ElteN378 nominal concentration such that $C_E < C$, and [FS] is the concentration of the FKBP12-α-syn complex at equilibrium. In Eq. 1 we have tacitly assumed that:

i) all added ElteN378 is instantly bound to FKBP12;[1]

ii) the concentration of the species [F_nS] with $n > 1$ are negligible when C=1 μM

iii) the equilibrium in the reaction FKBP12 + αsyn $\leftrightarrow$ FS is established at day zero.

We now define $f = (C - C_{\rm E})/C$ as the fraction of available (not inhibited) FKBP12 and $r = K_d/C$ as the ratio between the FKBP12-α-syn dissociation constant and the concentration of the 1:1 FKBP12/α-syn mixture. Solving Eq. 1 for $\phi \equiv [{\rm FS}]/C$, we obtain that the fraction of FKBP12 proline bound α-syn with respect to the initial concentration C is given by

$$\phi = \frac{1}{2}(1 + f + r)\left[1 - \left(1 - \frac{4f}{(1 + f + r)^2}\right)\right]^{1/2}. \tag{2}$$

ϕ in Eq. 2 depends hence on the reduced quantity $r = K_d/C$. In Figure S7 we show fraction of FKBP12 bound α-syn as a function of the fraction of available (not ElteN378-inhibited) FKBP12, ranging from 0 ($C_{\rm Elte} = C_F$) to 1 ($C_{\rm Elte} = 0$, no inhibitor). Assuming $r = 1$ (i.e. $K_d = C = 1$ μM), in the equimolar FKBP12-α-syn 1 μM solution the fraction of FKBP12 α-syn bound state is expected to be around 38%. Given the morphology of the experimental

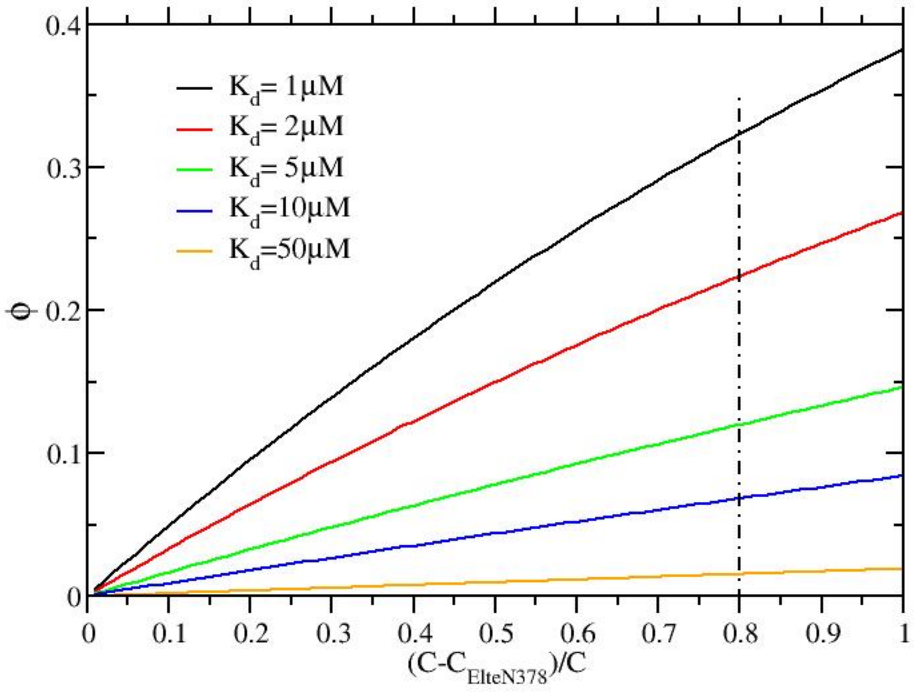

Figure S7: fraction of α-syn bound FKBP12 in 1 μM solution of 1:1 FKBP12/α-syn as a function of the effective FKBP12 concentration and for various values of the FKBP12-α-syn dissociation constant K_d. The vertical dashed line marks the effective FKBP12 concentration upon ElteN378 addition in the measurements.

aggregates in the 1:1 mixture with no inhibitor (see Figure 4 of the main paper, central upper panel), this fraction of FKBP12-monomer should be already beyond the transition FKBP12 concentration marking the fast dendrimeric growth according to our reduced CG model. Upon addition of the tight-binding sub-nanomolar FKBP12 inhibitor ElteN378[1] up to a 200 nM concentration, an equivalent amount of FKBP12 is deactivated from the 1:1 1μM solution of the α-syn FKBP12 mixture. As a result, the aggregation kinetics is significantly slower and dendrimeric growth is drastically reduced. The 0.2 μM ElteN378 addition reduces the fraction of FKBP12 α-syn bound complex to about 30%, i.e. near or right below the transition fraction occurring, according to our CG model, between $\phi = 0.3$ and $\phi = 0.5$.

Buried FKBP12 in aged α-syn aggregates: a guess from CG simulation Voronoi tessellation data

Table S1: Percentage of buried FKBP12 $c_{\rm buried}^{\rm FKBP12} = N_{\rm buried}^{\rm FKBP12}/N_{\rm total}^{\rm FKBP12}$ beads with respect to the total number of FKBP12 beads ($N_{\rm total}^{\rm FKBP12} \equiv 512 \times [{\rm FS}]/C$) and percentage of buried hydrophilic beads at position 2 in normal synuclein $c_{\rm buried}^{\rm M2-syn} = N_{\rm buried}^{\rm M2-syn}/N_{\rm total}^{\rm M2-syn}$ with respect to the total number of normal synuclein ($N_{\rm total}^{\rm M2-syn} = 512 - N_{\rm total}^{\rm FKBP12}$) as computed from the Voronoi volumes evaluations on the last configurations.

ϕ	$c_{\rm buried}$(FKBP12)	$c_{\rm buried}$(M2 − syn)
0.0	-	0.19 ±0.19
0.1	16.96 ±4.46	0.21 ±0.21
0.3	13.67 ±6.64	0 ±0.13
0.5	8.59 ±1.95	0.39 ±0.39
1.0	1.56 ±0.39	-

We finally examine the distribution of the FKBP12 seeds in the supramolecular structures at the end of the aggregation process. There is experimental evidence for a co-localization in brain cells of FKBP12 with the markers of pathology.[15,16] We recall that only very recently[17] FKBP12 was reported as one of the component of Lewy bodies in Parkinson's disease.[18] According to our aggregation model, the FKBP12 globular protein induces ramification of the supramolecular structure and is hence expected to be localized on a hydrophilic segment at the junction of the branches. The localization of FKBP12 in the aggregates is assessed from our configurational data using the Voronoi tessellation algorithm.[19] The final configurations are examined by computing in all samples the Voronoi volumes on the sub-ensemble of beads made by all the hydrophobic units and by all beads (mutated or not) in position 2 of an hydrophilic segment (see Figure S3). Voronoi volumes are evaluated in periodic boundary conditions using the minimum image convention and by closing the polyhedrons, when needed, by introducing 8 distant octahedral additional vertices placed at a distance of 10^4 nm from the central bead. In this manner, exposed beads have faces determined by the distant octahedral vertices, yielding extremely high Voronoi volumes. As discussed above, in the FKBP12-α-syn mixture, a fraction of these hydrophilic beads is replaced by hydrophobic

ones. As shown in Figure S8, the Voronoi volume distribution in the final state of pure α-syn

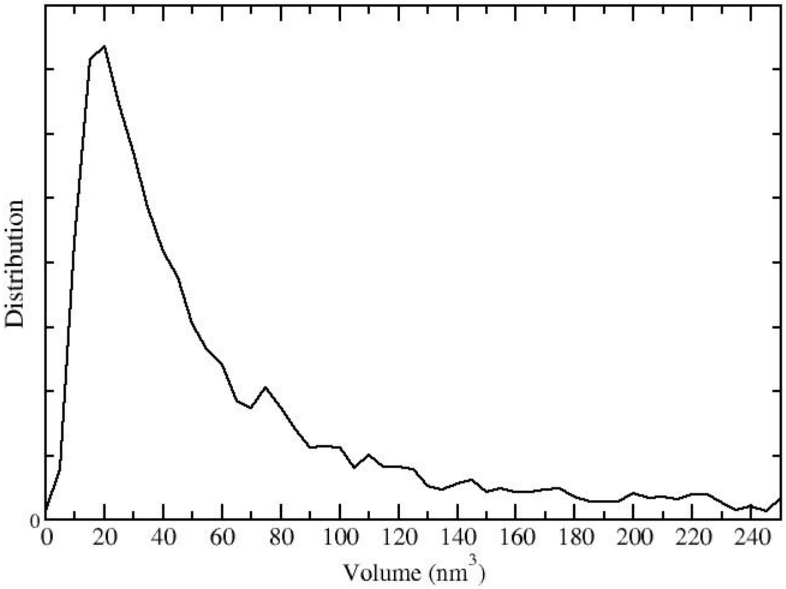

Figure S8: Voronoi volume distribution of the hydrophobic beads in pure α-syn. Volumes for exposed residues are in the range $V = 10^4 : 10^{12}$ nm.

peaks at about 20 nm^3 indicating a tight packing of the hydrophobic core of mature fibrils. Based on the distribution of Figure S8, we define a bead to be *buried* (i.e. within the interior of the hydrophobic core of the supramolecular aggregates) when its Voronoi volume is less than 50 nm^3. In Table S1 we show, for various values of the ratio $\phi = [\mathrm{FS}]/C]$, the results for the fraction of buried FKBP12 beads with respect to the total number of FKBP12 beads compared to the fraction of buried (non mutated) hydrophilic beads in position 2. The FKBP12 beads are clearly much more buried, on the average, with respect to the "normal" hydrophilic beads in position 2, that are basically solvent exposed in the vast majority with $c_{\mathrm{buried}}^{\mathrm{M2-syn}} \simeq 0$. Most of the non buried FKBP12 beads exhibit extremely large Voronoi volume indicating that these units are in general solvent exposed. We also note that, with increasing FKBP12 concentration, the fraction of buried FKBP12 beads decreases down to only 1.56% for $[\mathrm{FS}]/C] = 1$, i.e. in presence of an excess of available (not inhibited) FKBP12. Values

of $\phi > 0.38$ are in fact obtained only if the nominal concentration of FKBP12, C_F, is such that $(C_F - C_E)/C > 1$. So, in our FKBP12-α-syn equimolar 1 μM mixture, according to the data reported in Table S1, we expect that 1/3 of α-syn monomers are FKBP12-bound and that in these complexes only about the 10% of the FKBP12 is buried in the supramolecular cores. Hence, based on the simulation results, we estimate that in the 1:1 FKBP12-α-syn 1 μM mixture the amount of buried (non washable) FKBP12 in mature aggregates is in the nanomolar range (20:40 nM).

References

(1) Martina, M. R.; Tenori, E.; Bizzarri, M.; Menichetti, S.; Caminati, G.; Procacci, P. The Precise Chemical-Physical Nature of the Pharmacore in FK506 Binding Protein Inhibition: ElteX, a New Class of Nanomolar FKB12 Ligands. *J. Med. Chem.* **2013**, *56*, 1041–1051.

(2) Iwai, A.; Masliah, E.; Yoshimoto, M.; Ge, N.; Flanagan, L.; de Silva, H. R.; Kittel, A.; Saitoh, T. The precursor protein of non-Aβ component of Alzheimer's disease amyloid is a presynaptic protein of the central nervous system. *Neuron* **1995**, *14*, 467 – 475.

(3) Iljina, M.; Garcia, G. A.; Horrocks, M. H.; Tosatto, L.; Choi, M. L.; Ganzinger, K. A.; Abramov, A. Y.; Gandhi, S.; Wood, N. W.; Cremades, N.; Dobson, C. M.; Knowles, T. P. J.; Klenerman, D. Kinetic model of the aggregation of alpha-synuclein provides insights into prion-like spreading. *Proc Natl Acad Sci U S A* **2016**, *113*, E1206–E1215.

(4) Lyons, W.; Steiner, J. P.; Snyder, S. H.; Dawson, T. M. Neuronal Regeneration Enhances the Expression of the lmmunophilin FKBP-12. *J. Nuerosci.* **1995**, *15*, 2985–2994.

(5) Wärdehoff, M. M.; Bannach, O.; Shaykhalishahi, H.; Kulawik, A.; Schiefer, S.; Will-

bold, D.; Hoyer, W.; Birkmann, E. Single Fibril Growth Kinetics of α-Synuclein. *J. Mol. Biol.* **2015**, *427*, 1428–1435.

(6) Chelli, R.; Gervasio, F. L.; Procacci, P.; Schettino, V. Inter-residue and solvent-residue interactions in proteins: A statistical study on experimental structures. *Proteins: Structure, Function, and Bioinformatics* **2004**, *55*, 139–151.

(7) Procacci, P.; Paci, E.; Darden, T.; Marchi, M. ORAC: A Molecular Dynamics Program to Simulate Complex Molecular Systems with Realistic Electrostatic Interactions. *J. Comp. Chemistry* **1997**, *18*, 1848–1862.

(8) Marsili, S.; Signorini, G.; Chelli, R.; Marchi, M.; Procacci, P. ORAC: a molecular dynamics simulation program to explore free energy surfaces in biomolecular systems at the atomistic level. *J. Comp. Chem.* **2010**, *31*, 1106–1116.

(9) Procacci, P. Orac 6. 2017; Orac 6.1 can be downloaded under the GPL license from the internet address `http:/http://www.chim.unifi.it/orac`.

(10) Sabaté, R.; Rodriguez-Santiago, L.; Sodupe, M.; Saupe, S. J.; Ventura, S. Thioflavin-T excimer formation upon interaction with amyloid fibers. *Chem. Commun.* **2013**, *49*, 5745–5747.

(11) Sweers, K. K. M.; van der Werf, K. O.; Bennink, M. L.; Subramaniam, V. Atomic Force Microscopy under Controlled Conditions Reveals Structure of C-Terminal Region of α-Synuclein in Amyloid Fibrils. *ACS Nano* **2012**, *6*, 5952–5960.

(12) Holt, D. A.; Konialian-Beck, A. L.; Oh, H. J.; Yen, H. K.; Rozamus, L. W.; Krog, A. J.; Erhard, K. F.; Ortiz, E.; Levy, M. A.; Brandt, M.; Bossard, M. J.; Luengo, J. I. Structure-activity studies of synthetic FKBP ligands as peptidyl-prolyl isomerase inhibitors. *Bioorg. Med. Chem. Lett.* **1994**, *4*, 315–320.

(13) Bizzarri, M.; Tenori, E.; Martina, M. R.; Marsili, S.; Caminati, G.; Menichetti, S.; Procacci, P. New Perspective on How and Why Immunophilin FK506-Related Ligands Work. *J. Phys. Chem. Lett.* **2011**, *2*, 2834–2839.

(14) Burkhard, P.; Hommel, U.; Sanner, M.; Walkinshaw, M. The discovery of steroids and other novel {FKBP} inhibitors using a molecular docking program1. *J. Mol. Biol.* **1999**, *287*, 853 – 858.

(15) Avramut, M.; Achim, C. Immunophilins and their ligands: insights into survival and growth of human neurons. *Physiol. Behav.* **2002**, *77*, 463–468.

(16) Gerard, M.; Deleersnijder, A.; Daniels, V.; Schreurs, S.; Munck, S.; Reumers, V.; Pottel, H.; Engelborghs, Y.; Van den Haute, C.; Taymans, J.-M.; Debyser, Z.; Baekelandt, V. Inhibition of FK506 Binding Proteins Reduces α-Synuclein Aggregation and Parkinson's Disease-Like Pathology. *J. Neurosci.* **2010**, *30*, 2454–2463.

(17) Honjo, Y.; Ayaki, T.; Horibe, T.; Ito, H.; Takahashi, R.; Kawakami, K. FKBP12-immunopositive inclusions in patients with α-synucleinopathies. *Brain Res.* **2018**, *1680*, 39 – 45.

(18) Shults, C. W. Lewy bodies. *Proc. Natnl. Acad. Sci. USA* **2006**, *103*, 1661–1668.

(19) Procacci, P.; Scateni, R. A general algorithm for computing Voronoi volumes: Application to the hydrated crystal of myoglobin. *International Journal of Quantum Chemistry* **1992**, *42*, 1515–1528.
